# Association study of genetic variants at newly identified lipid gene *TRIB1* with coronary heart disease in Chinese Han population

**DOI:** 10.1186/s12944-015-0043-0

**Published:** 2015-05-19

**Authors:** Long Wang, Jinjin Jing, Qianxi Fu, Xiaojun Tang, Li Su, Shishi Wu, Ge Li, Li Zhou

**Affiliations:** Department of Epidemiology, the Innovation Center for Social Risk Governance in Health, School of Public Health and Management, Chongqing Medical University, Chongqing, 400016 China; Department of Cardiology, the Second Affiliated Hospital of Chongqing Medical University, Chongqing, 400010 China; Department of Nutrition and Food Hygiene, School of Public Health and Management, Chongqing Medical University, Chongqing, 400016 China; Department of Epidemiology, School of Public Health and Management, Chongqing Medical University, Chongqing, 400016 China

**Keywords:** Genetic variant, Lipid, Coronary heart disease

## Abstract

**Background:**

Recent genome-wide association studies (GWAS) have identified the variants near *TRIB1* gene affecting blood lipid levels. However, the association between the reported variants and risk of coronary heart disease (CHD) was not confirmed.

**Methods:**

We conducted two independent case–control studies. The first study consisted of 300 CHD patients and 300 controls and the second study had 1,332 CHD patients and 2,811 controls. The genotypes of two variants rs3201475 and rs17321515 in *TRIB1* were determined by TaqMan assay. The dual-luciferase reporter assay was performed for evaluating the function of the SNP rs3201475.

**Results:**

The statistical analysis indicated that single nucleotide polymorphism (SNP) rs17321515 was replicated to be associated with triglyceride (TG) level, which was also significantly associated with CHD risk when using the stratified analysis after adjusting for conventional risk factors. Compared with *GG* genotype, *AA* carriers of SNP rs17321515 had higher risk in males (odds ratio (OR) = 1.28, 95 %CI = 1.01–1.61; *P* = 0.03) and smokers (OR = 1.41, 95%CI = 1.09–1.88; *P* = 0.01). We did not find significantly association between genotypes of rs3201475 and CHD risk. In addition, no significant difference was found in the luciferase activity assay of SNP rs3201475.

**Conclusions:**

Our findings indicated that SNP rs17321515 is significantly associated with plasma TG level and the increasing risk of CHD among males and smokers in Chinese, whereas there is no positive association between SNP rs3201475 and CHD risk. Smoking could modify the effects of *TRIB1* on CHD risk.

## Introduction

Coronary heart disease (CHD) is the leading cause of death worldwide [1]. World Health Organization (WHO) estimated that more than 700,000 people died from CHD each year. Recent genome-wide association studies (GWAS) have identified SNPs at several loci such as chromosomes 1p13, 3q22, 6p24, 9p21 and 15q22 that are associated with risk of CHD [2–4]. Meanwhile, a few loci were also shown to be strongly associated with plasma lipid levels, reinforcing the close mechanistic association between the variability in lipid levels and CHD risk.

*TRIB1* gene was firstly identified as a lipid-associated gene by Kathiresan through GWAS in the European population [2]. Then some other GWAS confirmed the association between *TRIB1* and lipids levels. Kathiresan *et al.* identified that the variants in *TRIB1* gene were associated with plasma triglyceride (TG) level [2]. Aulchenko *et al.* found that single nucleotide polymorphism (SNP) rs17321515 in *TRIB1* gene was associated with plasma total cholesterol (TC) level [3]. Recently, we conducted a GWAS of lipid levels in Chinese Han population and the data from SNP rs17321515 in *TRIB1* confirmed the association previously reported in Europeans [5].

Blood lipid levels have been consistently associated with risk of CHD. However, the relationship of *TRIB1* and CHD risk was indefinite. Meanwhile, the real functional SNP in *TRIB1* was not identified in previous studies. To further establish the associations between the genetic variant in *TRIB1*, lipid levels and risk of CHD and to improve understanding of the mechanisms underlying susceptibility to CHD, we conducted two independent CHD case–control studies in Chinese Han populations to test two SNPs rs17321515 and rs3201475 in *TRIB1* gene. SNP rs3201475, in the 5′ untranslated region (UTR) of *TRIB1*, was predicted to be a transcription factor binding site (TFBS) according to the transcription factor prediction tools: SNP Function Prediction and the JASPAR database. The prediction results showed that the SNP rs3201475 might have regulatory function in *TRIB1*. So we also examined whether the SNP rs3201475 affected gene expression by performing a reporter gene luciferase activity assay in two types of cell lines.

## Materials and methods

### Study populations

We performed two independent case–control analyses. The first population group consisted of 300 patients with CHD and 300 age- and sex-frequency matched healthy control subjects. The second population group consisted of 1,332 CHD cases and 2,811 controls. Patients of the former were consecutively recruited from three hospitals in Chongqing city between March 2012 and May 2013, and the latter were from hospitals in Wuhan and Chongqing cities between May 2008 and October 2013. The inclusion criteria for CHD were either: 1. the presence of a stenosis ≥ 50 % in at least one of the major segments of coronary arteries (the right coronary artery, left circumflex, or left anterior descending arteries) on coronary angiography; 2. based on World Health Organization criteria in terms of elevations of cardiac enzymes, electrocardiographic changes and clinical symptoms [6]; 3. a documented history of coronary artery bypass graft or percutaneous coronary intervention. Patients with congenital heart disease, cardiomyopathy and valvular disease were excluded. After cases were diagnosed with CHD, they were interviewed in person by a trained interviewer within 3 days. The control subjects, residing in the same communities as the cases, were determined to be free of CHD and peripheral atherosclerotic arterial disease by medical history, clinical examinations, and electrocardiography. Subjects with severe liver and/or kidney disease were excluded. Medical history, family history of CHD among first degree relatives, medication use, home environment, and lifestyle factors were obtained through questionnaire interview.

Subjects were classified as smokers and nonsmokers. Those who had smoked less than 100 cigarettes in the lifetime were defined as nonsmokers; otherwise, they were defined as smokers. The smokers were categorized into groups of cigarettes per day: < 20 and ≥20. BMI was calculated as weight in kilograms divided by the square of height in meters. All subjects of the two populations gave written consent after receiving a full explanation of the study. The Ethics Committee of Chongqing Medical University approved the present study, and written informed consent was obtained from all subjects.

### Determination of lipid levels

The plasma TC, TG, LDL and HDL levels were measured by the ARCHITECT Ci8200 automatic analyzer (ABBOTT Laboratories. Abbott Park, Illinois, U.S.A.) using the Abbott Diagnostics reagents according to the manufacturer’s instructions.

### Genotyping

Fasting venous blood was collected in 5-ml EDTA tubes, and genomic DNA was isolated with a Puregene kit (Gentra Systems, Inc., Minneapolis, MN, USA). Genotyping was performed with TaqMan SNP allelic discrimination by means of an ABI 7900HT (Applied Biosystems, Foster City, CA, USA), in 384-well format. The TaqMan Assay kit was purchased from Applied Biosystems (Foster City, CA, USA). It included the forward target-specific polymerase chain reaction (PCR) primer, the reverse primer, and the TaqMan MGB probes labeled with two special dyes: FAM and VIC. PCR reactions were carried out in reaction volume of 5 μl containing 5 ng DNA, 2.5 μl 2 × Taqman universal PCR Master MixNo AmpErase UNG (Applied Biosystems, Foster City, CA, USA), 0.125 μl 40 × Assay MIX. PCR conditions included 95 °C for 10 min followed by 40 cycles of 15 s at 92 °C and 1 min at 60 °C. Two blank controls (DNA hydration) and two replicate quality control samples were included in each 384-well format, and two replicate samples were genotyped with 100 % concordance. Automatic allele calling, with the default settings (the quality value of auto caller ≥95.0), was carried out by ABI 7900HT data collection and analysis software version 2.2.1 (SDS 2.2.1).

### Reporter plasmid construct

A 1,929 bp-sized fragment (from −1316 to +613) which includes the promoter and 5′ UTR of the *TRIB1* gene was polymerase chain reaction (PCR) amplified using either +164 T (rs3201475) homozygous or +164 C homozygous genomic DNA as a template and the following primers: forward primer: 5′– CGG***GGTACC***GGTGCCCAGGGACTCCAAAC −3′; the italic bold characters represent the KpnI site, reverse primer: 5′– CCC***AAGCTT***TCATGGCAGAGCGCACCGGA −3′; the italic bold characters denote the HindIII site. PCR conditions were as follows: Initial denaturation for 3 min at 95 °C, 30 cycles of denaturing at 98 °C for 40 s, annealing at 66 °C for 15 s and extension at 72 °C for 5 min. PCR products were purified using High pure PCR product purification Kit1 (Roche, Switzerland). The purified PCR product was sub-cloned into the KpnI–HindIII site of the pGL3-Basic luciferase reporter vector (Promega, Madison, WI, USA) to generate the pGL3−+164 plasmid. Site-directed mutagenesis at the +164 C/T site of the pGL3−+164 plasmid was carried out using a Muta-direct sitedirected mutagenesis kit (Saibaisheng, Beijing, China) following the manufacturer’s protocol. This process generated new construct with the +164 C/T site changed to +164 T/C site. These mutations were confirmed by DNA sequencing.

### Cell culture and luciferase assays

HepG2 and HeLa cells were maintained in Dulbecco’s modified Eagle’s medium (Gibco BRL, USA) supplemented with 10 % fetal bovine serum, penicillin (100 U/ml), and streptomycin (100 U/ml). Each constructed pGL3−+164 plasmid was transfected into HepG2 and HeLa cells with the pRL-SV40 plasmid (Promega, USA) by using Lipofectamine 2000 (Invitrogen, USA). Firefly luciferase and renilla luciferase activities were sequentially measured by a luminometer 48 h after transfection, utilizing a Dual-Luciferase reporter assay system (Promega, USA). Results were expressed as relative light units of firefly luciferase activity over relative light units of renilla luciferase activity. All experiments were performed in triplicate and repeated three times.

### Statistical analysis

Each continuous trait (TC, TG, LDL and HDL) was tested for normality and TG values were log-transformed. Continuous variables were reported as mean ± standard deviation (SD). Normal distribution of data was analyzed using the Kolmogorov-Smirnov normality test. Data with a normal distribution were compared by Student’s *t*-test, and those with unequal variance or without a normal distribution were analyzed by a Mann–Whitney rank sum test. Categorical values were compared by the chi-square test, which was also used to test for deviation of genotype distribution from Hardy-Weinberg equilibrium. The association between SNP and CHD risk was estimated by computing odds ratios (ORs) and 95 % confidence intervals (CIs) from the multivariate logistic regression analyses. The effects of genotypes on plasma lipid levels were assessed by multiple linear regression models. The Haploview program was used to analyze pairwise linkage disequilibrium (LD) with data extracted from International HapMap Project [7]. The probability level accepted for significance was *P* < 0.05. All data analyses were carried out with the statistical analysis software package SPSS 16.0 (SPSS Inc., Chicago, IL, USA).

## Results

### General characteristics of the subjects

We successively performed two case–control studies in the Chinese Han populations. Population I consisting 300 CHD cases and 300 controls was from Chongqing city, whereas population II consisting 1,332 CHD cases and 2,811 controls was from Wuhan and Chongqing cities. As shown in Table 1, in both of the populations, the age and sex were frequency matched between the cases and controls (*P* > 0.05). The proportion of smokers was significantly higher in cases than in controls (32 % *vs.* 21.3 % and 62 % *vs.* 50 %, respectively). In population I, only the lower high-density lipoprotein cholesterol (HDL) was observed in cases than in controls, whereas the higher fasting glucose, TC, low-density lipoprotein cholesterol (LDL) and lower HDL were observed in cases than in controls in population II. The proportion of subjects reported taking cholesterol-lowering medications such as statins and fibrates in the whole cases and controls were 17.4 % and 0.2 %, respectively.

**Table 1:** General characteristics of CHD patients and controls

Variable	Population I			Population II		
	Cases (*N* = 300)	Controls (*N* = 300)	*P*	Cases (*N* = 1332)	Controls (*N* = 2811)	*P*
Sex, m/f, (%)	163/137 (54.3/45.7)	160/140 (53.3/46.7)	0.81	969/363 (72.7/27.3)	2078/733 (73.9/26.1)	0.42
Age, mean ± SD	68.8 ± 10.3	67.6 ± 15.0	0.24	60.8 ± 10.0	61.2 ± 9.1	0.13
Body mass index(kg/m^2^)	23.8 ± 3.1	22.7 ± 4.6	0.12	24.5 ± 3.6	24.3 ± 3.5	0.25
Smoking, no/yes, (%)	204/96 (68.0/32.0)	236/64 (78.7/21.3)	<0.01	506/826 (38.0/62.0)	1406/1405 (50.0/50.0)	<0.01
Fasting glucose(mmol/L)	6.9 ± 2.8	7.2 ± 4.2	0.46	6.6 ± 3.5	5.3 ± 2.0	<0.01
Total Cholesterol(mmol/L)	4.3 ± 1.3	4.4 ± 1.2	0.21	4.7 ± 1.1	4.4 ± 1.0	<0.01
Triglyceride(mmol/L)	1.7 ± 1.5	1.5 ± 1.0	0.15	1.7 ± 1.2	1.6 ± 1.3	0.81
High-density lipid(mmol/L)	0.9 ± 0.3	1.1 ± 0.3	<0.01	1.0 ± 0.4	1.2 ± 0.3	<0.01
Low-density lipid(mmol/L)	2.5 ± 1.0	2.6 ± 0.9	0.69	2.7 ± 0.9	2.6 ± 0.8	<0.01

### Relation of the two SNPs in *TRIB1* and CHD risk

The observed genotype frequencies of the SNPs rs17321515 and rs3201475 were in Hardy-Weinberg equilibrium among the controls (*P* = 0.97 and 0.46, respectively). In population I, compared with *CC* genotype of rs3201475, the ORs of *CT* and *TT* genotypes were 0.91 (95 % CI: 0.54–1.54; *P* = 0.72) and 2.00 (95 % CI: 0.48–8.25; *P* = 0.34). In multivariate analyses, after adjusting for conventional CHD risk factors such as age, gender, smoking, body mass index (BMI), blood pressure and blood glucose, none genotypes of rs3201475 had an association with CHD risk. In population II, after adjusting for conventional CHD risk factors such as age, gender, smoking, BMI, blood pressure and blood glucose, the *AA* genotype of rs17321515 had significant associations with CHD (OR = 1.58, 95 % CI: 1.13–2.20, *P* = 0.01) (Table 2). We further conducted stratified analysis for the SNP rs17321515. Compared with *GG* carriers, *AA* carriers of SNP rs17321515 had higher risk in males (OR = 1.28, 95 % CI 1.01–1.61; *P* = 0.03) and smokers (OR = 1.41, 95 % CI 1.09–1.88; *P* = 0.01), especially in heavy smokers (≥20 cigarettes/day) (OR = 1.53, 95 % CI: 1.17–2.01; *P* = 0.002) (Table 3).

**Table 2:** Association of two SNPs in TRIB1 with CHD cases and controls in the Chinese Han populations

Genotypes	Cases n(%)	Controls n(%)	Crude OR (95%CI)	*P*-value	Adjusted OR^a^ (95%CI)	*P*-value
rs3201475 C > T						
CC	212 (70.67)	202 (67.33)	1.00 (referent)		1.00 (referent)	
CT	83 (27.67)	86 (28.67)	1.09 (0.76–1.56)	0.65	0.91 (0.54–1.54)	0.72
TT	5 (1.67)	12 (4.00)	2.52 (0.87–7.28)	0.09	2.00 (0.48–8.25)	0.34
rs17321515 G > A						
GG	429 (32.2)	936 (33.3)	1.00 (referent)		1.00 (referent)	
GA	638 (47.9)	1388 (49.4)	1.00 (0.86–1.16)	0.97	1.00 (0.77–1.29)	0.99
AA	265 (19.9)	487 (17.3)	1.19 (0.99–1.45)	0.06	1.58 (1.13–2.20)	0.01

^a^ Data were calculated by logistic regression analysis with adjustment for age, sex, smoking, BMI, blood pressure, blood glucose

**Table 3:** Stratification analysis for association between SNP rs17321515 genotypes and risk of CHD

		OR (95 % CI)^a^			*P* ^a^
		*GG*	*GA*	*AA*	
Gender	Male	1.00	1.03(0.86–1.23)	1.28(1.01–1.61)	0.03
	Female	1.00	0.94(0.69–1.27)	1.02(0.69–1.51)	0.86
Age(years)	≤60	1.00	1.22(0.96–1.55)	1.29(0.95–1.76)	0.11
	>60	1.00	0.86(0.70–1.06)	1.15(0.88–1.49)	0.16
BMI(kg/m^2^)	<25	1.00	0.89(0.72–1.09)	1.20(0.93–1.54)	0.15
	≥25	1.00	1.19(0.93–1.53)	1.21(0.87–1.68)	0.16
Smoke status	Non-smokers	1.00	0.96(0.75–1.21)	0.96(0.71–1.32)	0.82
	Smokers	1.00	1.03(0.84–1.26)	1.41(1.09–1.88)	0.01
	<20 cigarettes/day	1.00	0.97(0.72–1.30)	1.02(0.69–1.51)	0.95
	≥20 cigarettes/day	1.00	1.06(0.84–1.33)	1.53(1.17–2.01)	0.002
Alcohol consumption	Non-drinkers	1.00	1.03(0.83–1.28)	1.25(0.95–1.65)	0.24
	Drinkers	1.00	1.03(0.72–1.47)	1.16(0.73–1.83)	0.82
Diabetes	Yes	1.00	1.10(0.81–1.50)	1.32(0.90–1.94)	0.37
	No	1.00	0.96(0.77–1.20)	1.03(0.77–1.38)	0.84
Hypertension	Yes	1.00	1.03(0.80–1.34)	1.18(0.84–1.64)	0.62
	No	1.00	0.99(0.76–1.30)	1.20(0.85–1.69)	0.49

^a^ORs and *P* values were obtained from a logistic regression model with adjustment for age, sex, smoking, BMI, blood pressure, blood glucose

### Association between SNP rs17321515 in *TRIB1* and TG level

**Fig. 1:**
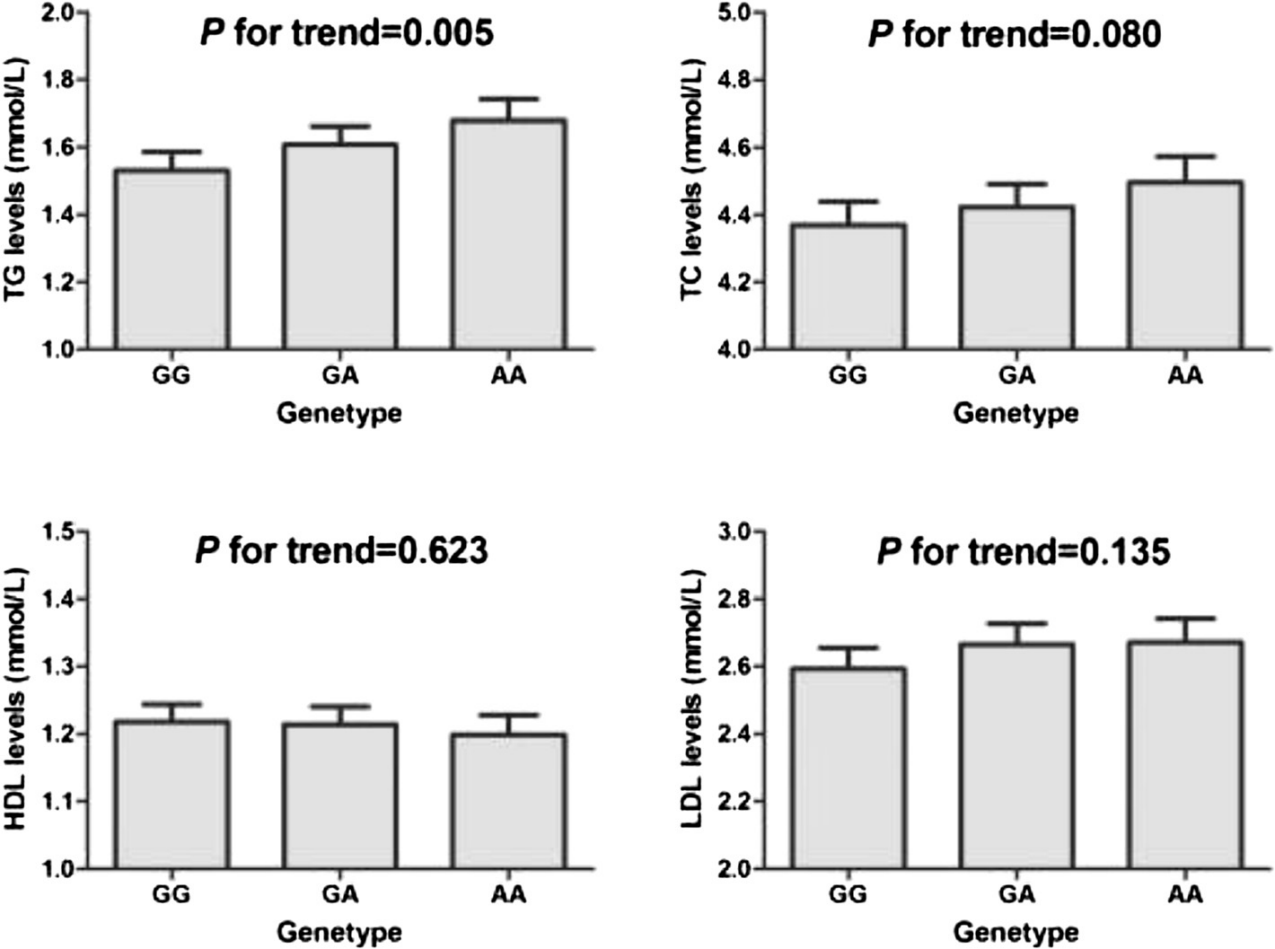
Associations between SNP rs17321515 in *TRIB1* and lipid levels in Chinese. The genotype represents three genotypes of the SNP rs17321515. The height of the bars is the mean values of lipid levels with a specific genotype. The error bars are Means ± SD of lipid levels in each group. *P* for trend was calculated by the multiple linear regression analyses adjusted for age, gender, BMI and smoke status

Statistical analysis was performed for the association between the SNP rs17321515 and plasma TC, TG, LDL and HDL levels in the control subjects in population II. General linear model analysis showed no significant for TC, LDL and HDL levels under an additive, dominant, or recessive model. Interestingly, we observed significant associations between rs17321515 and increased TG level (*P* for trend = 0.005) in additive model in 2,811 control subjects. Results of the multiple linear regression analyses adjusted for age, gender, BMI and smoke status were shown in Fig. 1. Each minor allele *A* increased TG level from 1.56 to 1.69 mmol/L (*GG* = 1.56 mmol/L; *GA* = 1.65 mmol/L; *AA* = 1.69 mmol/L).

### Reporter gene luciferase activity assay

**Fig. 2:**
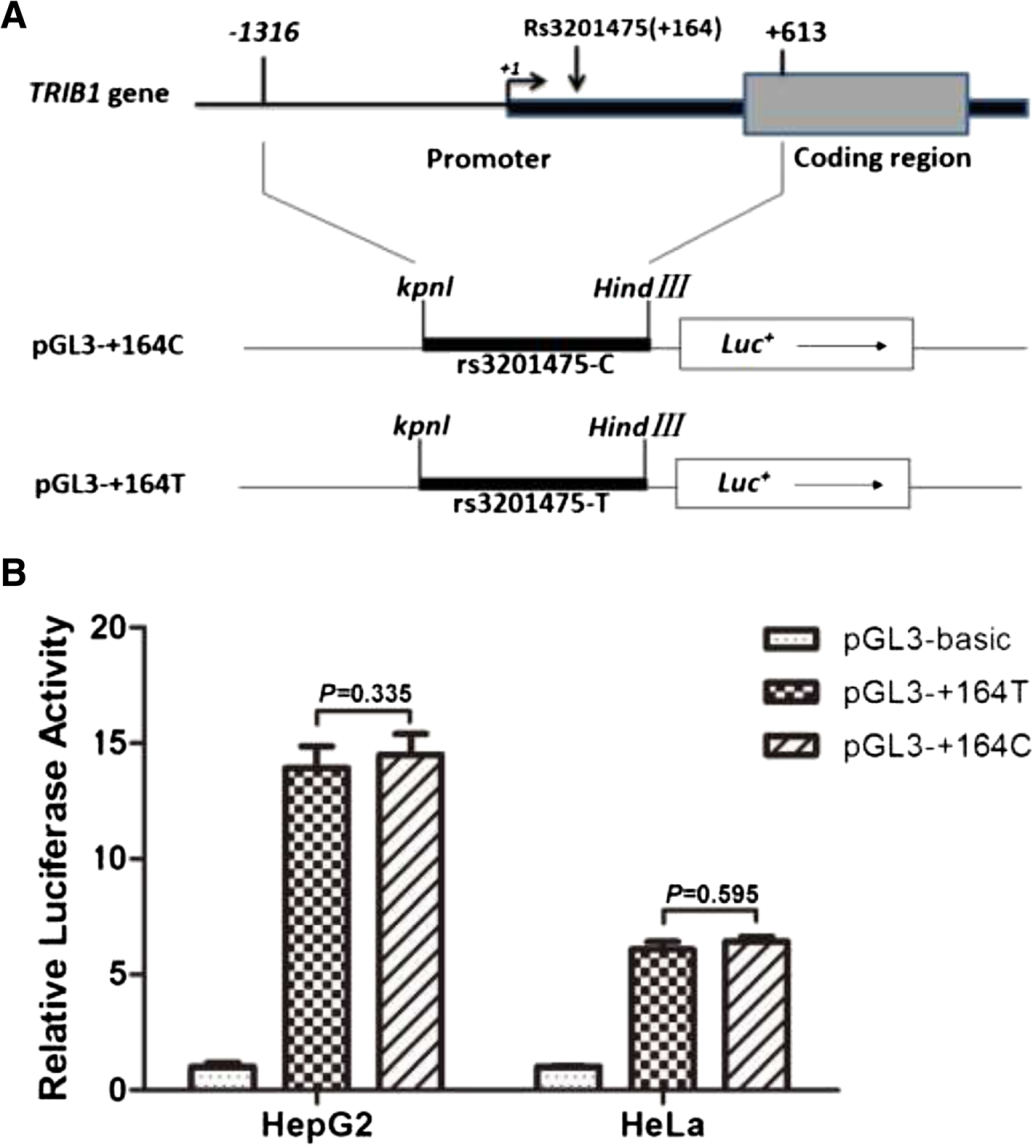
The construction of experimental plasmid vectors and the relative luciferase activity values. **a** The construction of experimental plasmid vectors. A 1,929 bp-sized of *TRIB1* fragment including rs3201475 C or T allele was inserted into the pGL3-basic vector, and pRL-SV40 vector was used in combination with other reporter vectors to co-transfect the HepG2 and HeLa cell lines. **b** The relative luciferase activity values of three transfection vectors in the HepG2 and HeLa cell lines. Values were obtained as Means ± SD

The *TRIB1* promoter and 5′ UTR with a length of 1,929 bp were amplified. Figure 2 showed the construction of pGL3−+164C and pGL3−+164T plasmids, indicating the difference at only one locus. The relative luciferase activities were obtained by dividing the firefly luciferase by renilla luciferase and the values were obtained as mean ± SD. The relative luciferase activity values of the three transfection vectors in two types of cell lines were shown in Fig. 2. *T*-test analysis showed that the relative luciferase activities were significantly higher in pGL3−+164C and pGL3−+164T compared with that in pGL3-basic. However, there were no significant differences between pGL3−+164C and pGL3−+164T in the observed luciferase activities in both HepG2 and HeLa cell lines.

## Discussion

In this study, we conducted two independent case–control studies to investigate whether a new genetic susceptibility locus *TRIB1* was associated with lipid levels and involved in the development of CHD. However, only one SNP rs17321515 near *TRIB1* had modestly association with CHD susceptibility in Chinese Han population. Another SNP rs3201475 which was in the 5′ UTR region of *TRIB1* had no significant association in case–control study and luciferase assays.

We identified the SNP rs17321515 in *TRIB1* that influence plasma TG level in Chinese, and also provided evidence that this SNP was significantly associated with CHD risk in a large independent case–control study in Chinese. Our results showed that genotype *AA* of the SNP rs17321515 was associated with increased risk of CHD among males and smokers. It is possible that genetic factors would exert a greater influence in males, and smoking may exacerbate the influence of the genetic factors. However, there was no significant relationship between the SNP rs3201475 and CHD susceptibility in Chinese Han population. In addition, no significant difference between C and T allele transcriptional activity was observed when using HepG2 and HeLa cell lines.

Significant associations of the SNP rs17321515 at 8q24 near *TRIB1* with lipid levels have been replicated in several studies. The minor *G* allele at this SNP was associated with lower TG, lower LDL and higher HDL levels. Kathiresan *et al.* identified SNP rs17321515 was strongly associated with TG and was also associated with LDL and HDL levels [2]. Wang *et al.* found that *TRIB1* had significant high risk for severe hypertriglyceridemia [4]. Garcia-Rios *et al.* reported that rs17321515 was associated with familial hypercholesterolemia in Spanish population [8]. Recently, our GWAS also showed that SNP rs17321515 in *TRIB1* was significantly associated with TG level in Chinese Han population [5]. However, few studies have examined the associations between the SNPs in *TRIB1* and CHD risk. Willer *et al.* reported that SNP rs17321515 in *TRIB1* which associated with increased TG level was also associated with increased risk of cardiovascular disease [9]. Recently, a Danish study replicated the association between another SNP rs2954029 in *TRIB1* and risk of ischemic heart disease in White descent in more than 71,000 individuals [10]. Also, in an Asian Malay study, Tai *et al.* found rs17321515 to be associated with an increased risk of cardiovascular disease [11], which is consistent with our results.

The frequency of the risk allele *A* of rs17321515 is different between the Chinese Han population and European population. The data from the HapMap showed that the rs17321515-*A* allele differs from 0.305 in Europeans to 0.178 in Han Chinese. The SNPs which were strongly associated lipids levels and CHD risk lie considerably downstream of *TRIB1* gene, and the most significantly SNP rs17321515 is about 44 kb downstream of it.

Rs3201475, a polymorphism in 5′ UTR of the *TRIB1* gene, predicted to be a TFBS with the minor allele frequency (MAF) to be 0.122 in Chinese Han population according to the NCBI database. However, there was no literature had studied on it. In our study, no significant association was observed between the SNP rs3201475 and CHD susceptibility in Chinese Han population and no significant difference between C and T allele transcriptional activity levels in both HepG2 and HeLa cell lines, suggesting the variant is not CHD causal variant. Considering the possible causes, the lipid-associated SNPs in *TRIB1* identified by GWAS were located 30 kb downstream from *TRIB1* and this gene has not yet been identified as an expression trait locus (eQTL). It suggested that the genomic effects of this locus may not be limited to alterations in expression of the *Trib1* protein [9]. Furthermore, the binding of transcription factors to a specific DNA sequence is dependent on various intracellular and extracellular stimuli within a cell. Therefore, although some transcription factors are predicted to bind to these sequences, this does not take into account *in vivo* conditions such as chromatin structure, and are hence not ideal representations of what occurs in the cell [12].

**Fig. 3:**
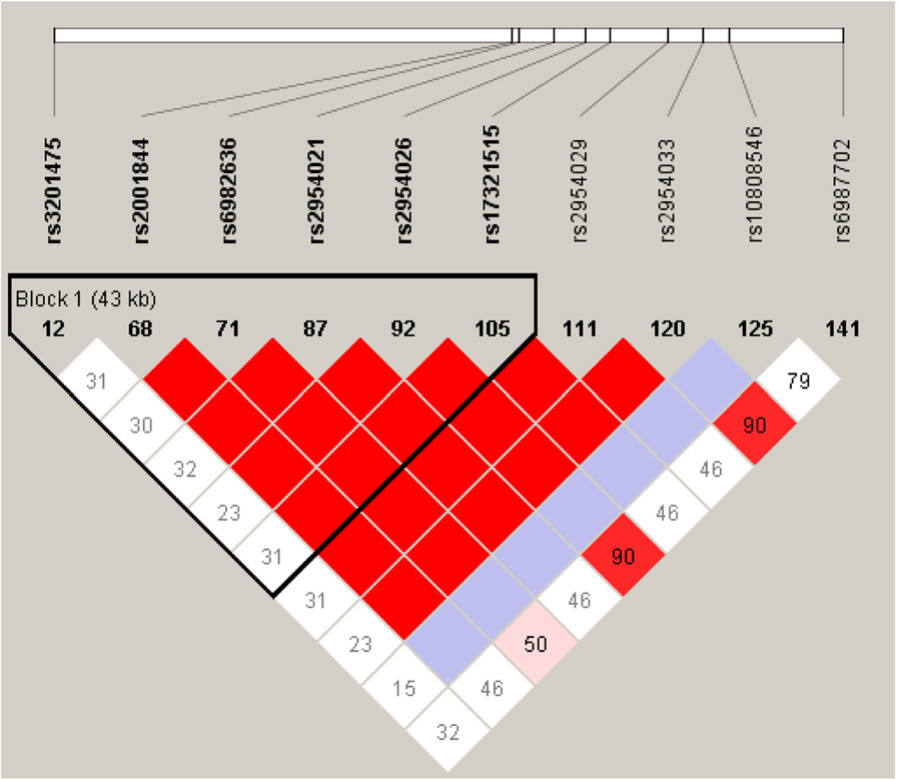
Linkage disequilibrium patterns in *TRIB1* gene. The pair-wise LD between the SNPs is indicated by diamonds shaded in white-gray-red, which show the range of the LD matrix from D’ = 0 in white to D’ = 1 in red

To gain insight into the linkage disequilibrium (LD) pattern of SNPs rs17321515, rs3201475 and other SNPs with strong evidence of association, the data for these SNPs were extracted from HapMap datasets for LD analysis. A battery of significant SNPs, rs2001844, rs6982636, rs2954021, rs2954026, rs17321515, rs2954029, and rs2954033, virtually represented the same statistical association signal, suggesting those associated SNPs were in strong LD in Chinese Han population. Notably, the SNP rs3201475, locating 40 kb distance from the region including rs17321515, displayed poor LD with the multiple associated SNPs with the r^2^ less than 0.02 (Fig. 3).

*TRIB1* encodes a G-protein-coupled receptor-induced protein, a member of the family of proteins that act as secondary messengers in the mitogen-activated protein kinases (MAPK)-related signaling cascade [13, 14] and may regulate lipid metabolism through this pathway [15]. It has also been suggested that *TRIB1* expression is regulated by inflammatory stimulation [16]. *TRIB1* controls chemotaxis and proliferation of smooth muscle cells in the arterial intima [17]. However, the exact mechanism of *TRIB1* in the development of atherosclerosis is still not known. It is interesting to note that smoking acts as modifiers of the association between *TRIB1* and CHD risk. Garcia-Rios *et al.* observed that the variants in *TRIB1* was only associated with familial hypercholesterolemia in smokers [8]. It has been demonstrated that nicotine induces MAPK dependent vascular smooth muscle cell migration resulting in the development of atherosclerosis and that cigarette smoking enhances MAPK activation [18, 19]. These findings suggest that the smoking-induced associations between *TRIB1* and CHD risk may involve MAPK.

Another study in Han and Mulao populations in China found that the lipid levels were associated with rs17321515 in males. Serum lipids were also associated with age, gender, BMI, blood pressure, blood glucose and alcohol consumption [20]. All of them were risk factors of the CHD and we adjusted these traditional factors in our study.

There is limitation of our study that must be acknowledged. The subjects used to test the association between the genotypes and CHD risk did not exclude the individuals who were receiving lipid-lowering medications. About 17.4 % CHD cases and 0.2 % controls in our study reported taking cholesterol-lowering medications, so we could not explore the association between the genotypes and lipid levels in all subjects. Fortunately, the very low frequency in control subjects (0.2 %) did not significantly affect the results of the association between the genotype and lipid levels. We identified the effect of genetics on lipid levels in the control subjects.

## Conclusion

In summary, SNP rs17321515 near *TRIB1* was associated with plasma TG level, and this SNP was significantly associated with increased risk of CHD in males and smokers in Chinese. Smoking could modify the effects of *TRIB1* on CHD risk. SNP rs3201475 in *TRIB1* showed no significant association with CHD risk. Further studies are needed to validate these findings and investigate potential mechanisms underlying the links between the variation of *TRIB1* gene and CHD risk.

### URLs

SNP Function Prediction, http://snpinfo.niehs.nih.gov/snpinfo/snpfunc.htm; JASPAR database, http://jaspar.genereg.net/cgi-bin/jaspar_db.pl; International HapMap Project, http://hapmap.ncbi.nlm.nih.gov/.
